# Evaluating the Efficacy of a Novel Titanium Cage System in ALIF and LLIF: A Retrospective Clinical and Radiographic Analysis

**DOI:** 10.3390/jcm14165814

**Published:** 2025-08-17

**Authors:** Ryan W. Turlip, Mert Marcel Dagli, Richard J. Chung, Daksh Chauhan, Richelle J. Kim, Julia Kincaid, Hasan S. Ahmad, Yohannes Ghenbot, Jang Won Yoon

**Affiliations:** 1 Department of Neurosurgery, University of Pennsylvania Perelman School of Medicine, Philadelphia, PA 19104, USA; 2 Department of Neurosurgery, University of Virginia School of Medicine, Charlottesville, VA 22903, USA; mbm7eu@virginia.edu; 3 Columbia College, Columbia University, New York, NY 10027, USA

**Keywords:** lumbar interbody fusion, degenerative spine disease, interbody cage, linear mixed-effects model, titanium cage

## Abstract

**Background/Objectives**: The success of lumbar interbody fusion depends on the implant design and the surgical approach used. This study evaluated the clinical and radiographic outcomes of lateral lumbar interbody fusion (LLIF) and anterior lumbar interbody fusion (ALIF) using a 3D-printed porous titanium interbody cage system. **Methods**: A retrospective, single-center review of 48 patients treated for degenerative lumbar spine disease was conducted. Patients underwent LLIF, ALIF, or a combination of both using a 3D-printed titanium cage system (J&J MedTech, Raynham, MA, USA). The Oswestry disability index (ODI) and Patient-Reported Outcomes Measurement Information System (PROMIS) metrics were assessed after 6 weeks, 3 months, 6 months, and 12 months. Linear mixed-effects models evaluated the pre- and post-operative differences. Fusion performance and complications were assessed using the Bridwell grading system over 24 months. **Results**: A total of 78 levels (62 LLIF and 16 ALIF) were analyzed. Fusion rates were 90.3% (56/62) for LLIF levels and 81.3% (13/16) for ALIF levels by the end of 12 months. ODI scores improved significantly after 3 months (MD −13.0, *p* < 0.001), 6 months (MD −12.3, *p* < 0.001), and 12 months (MD −14.9, *p* < 0.001). PROMIS Pain Interference scores improved after 3 months (MD −6.1, *p* < 0.001), 6 months (MD −3.4, *p* < 0.001), and 12 months (MD −5.8, *p* < 0.001). PROMIS Physical Function scores improved after 3 months (MD +3.4, *p* = 0.032) and 12 months (MD +4.9, *p* < 0.001). **Conclusions**: This novel interbody cage demonstrated high fusion rates, significant pain and function improvements, and a favorable safety profile, warranting further comparative studies.

## 1. Introduction

Anterolateral (ALIF) and lateral lumbar interbody fusion (LLIF) are retroperitoneal surgical approaches for achieving indirect decompression and stabilization of the lumbosacral spine for the treatment of degenerative spine disease (DSD) [1]. Both ALIF and LLIF approaches avoid the posterior spinal elements, manipulating the psoas muscles and the surrounding neurovascular anatomy to decompress affected neural structures, restore disc height, correct lumbar lordosis, and place interbody devices [2,3,4,5]. When compared to posterior (PLIF) and transforaminal lumbar interbody fusion (TLIF), ALIF and LLIF have been shown to be more effective in correcting lumbar lordosis [1,2,5]. However, achieving long-term fusion rates and implant longevity from surgical intervention are highly dependent on both the placement and properties of the interbody implant placed within the intervertebral space [6].

Emerging technologies within the past few decades have vastly broadened the range of interbody implants available for treating patients with lumbar DSD. Commonly utilized materials for synthetic interbody fusion devices include carbon fiber, titanium, and polyether ether ketone (PEEK) [7]. PEEK cages are widely favored by surgeons due to their bone-like elastic modulus, used to reduce stress shielding, and radiolucent properties that enhance visibility across imaging modalities [8,9]. In contrast, titanium cages offer osteoconductive benefits and can be engineered with porous designs that mimic trabecular bone, promoting direct integration into the bone itself [10]. The latest modifications of PEEK cages have introduced similar porous designs with titanium coatings that have been shown to have near-identical clinical outcomes as PEEK cages alone, albeit with limited data to conclude a significant advantage [11,12]. The selection of the appropriate interbody device, paired with the optimal bone graft material, is critical to maintaining the adequate neural decompression achieved during surgery and mitigating the risks of postoperative migration or subsidence.

More recently, the advent of nanoscale surface technology has significantly advanced osteogenesis in spinal fusion. The custom modification of 3D-printed porous titanium cages roughens the surface topography of each internal porous cell structure with micro- and nano-textured features to stimulate osteointegration. Previously, microroughened titanium surfaces have been shown to promote an osteoblastic environment to optimize fusion rates [13]. Current advances reveal that nanoscale surface technology can facilitate molecular-level interactions between the implant and host cells, to initiate osteoblastic differentiation via cell-membrane receptors [13,14,15,16]. Furthermore, the combined surface properties of microrough and nanostructured implants have been demonstrated, across in vitro studies, to act synergistically, thereby promoting long-term osseointegration and accelerating interbody fusion [15,16,17,18].

Novel interbody devices with a combined microrough and nanostructured porous titanium cage are being developed and offer promising osteogenic advantages in facilitating solid fusion constructs. This design aims to lower the risk of subsidence or migration and enhance patient outcomes. Nanostructure porous titanium cages are engineered to have a pore size of 700 µm, optimized for bony in-growth with an elastic modulus between that of cortical and trabecular bone, similar to the properties of PEEK cages [19]. Their porous interconnectivity also enhances the radiographic visibility of the endplate architecture, improving the assessment of bony fusion integrity both intra- and postoperatively. Despite these proposed advantages, the clinical outcome and efficacy of nanostructured titanium cages have not yet been described in the literature within surgical cohorts.

This study aimed to investigate the early post-operative clinical and radiographic outcomes of a novel microrough and nanostructured porous titanium ALIF and/or LLIF cages in the treatment of skeletally mature patients with DSD of the lumbar spine in a preliminary case series. Our study evaluated the patient-reported outcome measures (PROMs) at progressive time intervals, in addition to objective perioperative outcomes, to accurately capture the clinical timeline of cage integration.

## 2. Materials and Methods

### 2.1. Study Design

This retrospective, single-center study utilized the chart reviews of a consecutive series of patients. Information on the patients’ demographic, clinical, radiographic, and procedural characteristics at the time of surgery, as well as patient outcomes and complications during follow-up, was collected and analyzed. This study received approval as an exempt human subjects research study from the Institutional Review Board under 45 CFR 46.110 guidelines, since there was no patient contact as part of the study, and any data generated were extracted from the electronic medical records. The design and reporting of this study were supported by the following reporting structures: strengthening the reporting of observational studies in epidemiology (STROBE) and transparent reporting of a multivariable prediction model for individual prognosis or diagnosis + artificial intelligence (TRIPOD+AI) [20,21].

### 2.2. Data Source

The study analyzed data from 48 consecutive patients who underwent lumbar fusion surgery for degenerative spine disease at one or multiple levels from L2 to S1, incorporating the novel CONDUIT^TM^ Lateral System and ALIF System (J&J MedTech, Raynham, MA, USA). All procedures were performed by a single Fellowship-trained attending neurosurgical spine surgeon at a multi-hospital academic institution between January 2019 and October 2022. Among these, 32 patients underwent LLIF only, 12 underwent a combination of LLIF and ALIF, and 4 underwent ALIF only.

### 2.3. Sample Selection

Patients were identified through their medical records, based on surgical codes and implanted devices. The study included patients who met the following eligibility criteria.

Inclusion criteria:

- Adults aged 18 to 80 years, with lumbar degenerative spine disease requiring interbody fusion at one or more levels between L2 and S1.
- Patients treated with either an LLIF, an ALIF, or a combination of LLIF and ALIF devices, with supplemental fixation as necessary.

Exclusion criteria:

- Patients under 18 years or over 80 years of age at the time of surgery.
- Patients with lumbar disease from infections (e.g., tuberculosis) or of neoplastic, traumatic, or congenital etiology.
- Patients necessitating deformity correction.
- Patients with severe osteoporosis.

To minimize selection bias, all consecutive patients who met the inclusion and exclusion criteria were included in the analysis.

### 2.4. Preoperative and Procedural Characteristics

Key variables collected included patient demographics (age, sex, BMI, smoking status, primary diagnosis, and comorbid conditions) and surgical details (treated levels, approach type, the use of supplemental fixation, additional procedures, operative time, blood loss, length of hospital stay, and type of bone grafting material). All patients were operated on using a retroperitoneal approach, and the transpsoas approach was the one most commonly used. The decision to use supplemental posterior fixation and/or direct posterior decompression was based on individual patient pathology, with most patients receiving pedicle screw fixation in addition to interbody cage placement. Direct posterior decompression (e.g., laminectomy) was performed selectively when indirect decompression was deemed insufficient.

### 2.5. Clinical Outcomes

Clinical evaluations focused on neurological function, including assessments of sensory and motor status, reflexes, and gait, which were categorized as normal or abnormal. Subjective clinical variables included the Oswestry disability index (ODI), patient-reported outcomes measurement information systems (PROMIS) Depression, PROMIS Physical Function, and PROMIS Pain Interference metrics. Post-operative PROM timepoints were set after 6 weeks, 3 months, 6 months, and 12 months.

### 2.6. Radiographic Outcomes

Radiographic assessments included the evaluation of spinal fusion using the Bridwell interbody fusion grading system, wherein grades 1 and 2 indicated fusion and grades 3 and 4 represented non-union [22]. Additional radiographic outcomes included measurements of global and segmental lumbar lordosis, as well as vertebral disc height, calculated as the average of anterior and posterior disc heights. Complications such as implant subsidence, migration, and spondylolisthesis were also documented across a 24-month period. Standing lateral radiographs were used to measure segmental lumbar lordosis, disc height, and the rate of subsidence. Subsidence was classified using the following scale: Grade 0, 0–24% loss of postoperative disc height; Grade I, 25–49% loss; Grade II, 50–74% loss; and Grade III, 75–100% loss [23]. Radiographic fusion outcomes were assessed for patients with either (1) imaging follow-up at ≥12 months or (2) earlier documented evidence of fusion (Bridwell Grade 1 or 2). Patients with a <12-month follow-up and no earlier evidence of fusion were excluded from the fusion analysis. Fusion was assessed cumulatively, with satisfactory fusion status at the earliest confirmed timepoint carried forward to the final analysis. Patients with a follow-up beyond 12 months were included in this category, although imaging beyond 12 months was not universally available. As post-operative computed tomography (CT) scans are not routinely performed at our institution, radiographic outcomes were measured using X-rays [24,25]. CT scans were prioritized in those instances when they were available.

### 2.7. Adverse Events and Complications

Adverse events (AEs) were recorded at multiple timepoints, including intraoperative events, postoperative events prior to discharge, and during follow-ups after 6 weeks, 3 months, 6 months, 12 months, and 24 months when available. Revision surgeries were defined as any reoperation at the index levels whereby the implanted device was altered, repositioned, removed, or supplemented with additional devices, regardless of surgical approach. Reoperations included additional surgeries in the lumbar region that did not involve modifications to the implanted device.

### 2.8. Missing Data

Due to a high degree of missingness in PROMs at the 24-month timepoint, these data were excluded from statistical analyses. After excluding the 24-month PROMs, the overall proportion of missingness in PROMs and demographic variables across earlier timepoints was low, remaining below 5%. Missing PROMs data were imputed using a multiple imputation approach, employing a random forest machine learning methodology with a different random state adopted for each imputation to account for uncertainty. Other clinical and radiographic variables were reported through the 24-month follow-up period when available, with the number of missing observations at each timepoint being reported.

### 2.9. Statistics

PROMs were the primary outcome of this study and were analyzed using a linear mixed-effects model (LMM) to evaluate inter-group differences in the ODI, PROMIS Pain Interference, PROMIS Physical Function, and PROMIS Depression scores at pre-operative and post-operative timepoints of 6 weeks, 3 months, 6 months, and 12 months. The model incorporated baseline characteristics and included random intercepts. The estimated marginal means were calculated and averaged across covariates to provide adjusted estimates. All statistical analyses and figure generation processes were performed using Python (version 3.12.7, Python Foundation, Wilmington, DE, USA), integrated with R (version 4.4.2, R Foundation for Statistical Computing, Vienna, Austria) through the rpy2 package (version 3.5.16).

Descriptive statistics were used to summarize secondary outcomes across the overall sample and for individual cohorts—LLIF-only, LLIF combined with ALIF, and ALIF-only. Patient demographics, radiographic outcomes, and clinical measures were analyzed. For categorical variables, percentages and counts were reported, while continuous variables were described using means, medians, ranges, and standard deviations. Radiographic fusion outcomes were assessed for patients with at least 12 months of follow-up data or earlier documented fusion. Additional radiographic metrics, such as lumbar lordosis and intervertebral disc height, were evaluated across all levels treated with either LLIF or ALIF implants, regardless of surgical grouping.

## 3. Results

### 3.1. Study Population

A total of 48 consecutive patients were included in the analysis, with 78 spinal levels having been operated on (62 LLIF, 16 ALIF). Of these, 32 patients underwent LLIF only, 12 underwent a combination of LLIF and ALIF, and 4 underwent ALIF only. The mean age at surgery was 60.3 ± 10.5 years for the LLIF-only cohort, 62.3 ± 12.4 years for the combined cohort, and 42.8 ± 10.9 years for the ALIF-only cohort. Most patients were female (62.5% in the LLIF group, 58.3% in the combined group, and 75% in the ALIF group). The mean follow-up duration was 12.04 ± 5.78 months (range 1–25 months) for all patients (Table 1).

Most patients (75%) in the LLIF cohort underwent single-level fusion, while the ALIF cohort exclusively underwent single-level procedures. LLIF cages were most commonly implanted at L4/L5 and L3/L4 levels, while ALIF cages were primarily placed at L5/S1. Most patients (85.4%) received supplemental fixation with pedicle screws. The average estimated blood loss was 260.42 ± 235.02 mL, with the combined cohort experiencing the highest mean blood loss at 420.83 ± 229.09 mL. Most patients (72.9%) had a hospital stay of 5 days or fewer (Table 2).

### 3.2. Radiographic Outcomes and Adverse Events

**Figure 1:**
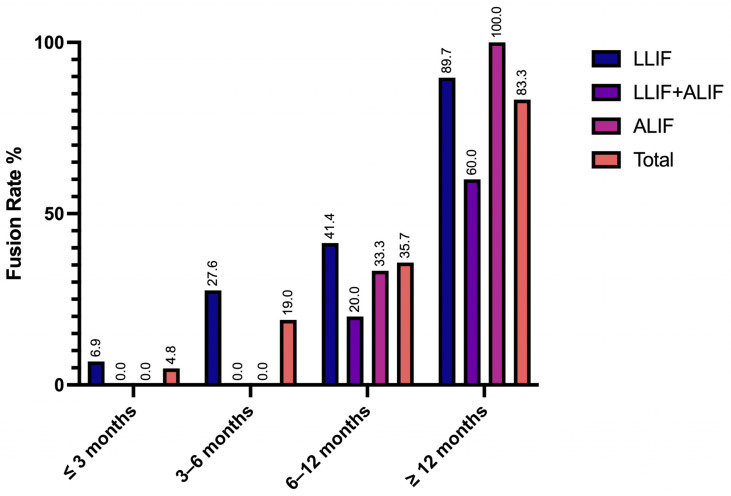
Fusion rates separated by cohort and post-operative follow-up intervals. Satisfactory fusion was defined as Bridwell interbody fusion grade 1 or 2, and was calculated cumulatively. The 3–6-month interval includes assessments performed > 3 and ≤6 months postoperatively; the 6–12-month interval includes assessments performed > 6 and ≤12 months postoperatively. The ≥12-month category represents the final cumulative fusion rate, reflecting the earliest time at which satisfactory fusion was confirmed for each patient and carried forward, with final follow-ups extending up to 25 months.

Radiographic fusion data were available for a total of 42 patients (29 LLIF, 10 combined, 3 ALIF) and were analyzed according to cohort. Among patients with at least 12-month follow-up data or a prior confirmed fusion status, 89.7% of the LLIF cohort, 60% of the combined cohort, and 100% of the ALIF cohort achieved fusion (Figure 1). Fusion rates per implanted level were 90.3% (56/62) for LLIF levels and 81.3% (13/16) for ALIF levels. The non-union rate at final follow-up was 9.4% in the LLIF cohort, 33.3% in the combined cohort, and none in the ALIF cohort. Radiographic complications included subsidence (LLIF—18.8%, ALIF—none, Combined—16.7%) and cage migration (LLIF—3.1%, ALIF—25.0%, Combined—16.7%).

Other radiographic data were collected, including global lumbar lordosis, segmental lumbar lordosis, disc height, and foraminal height measurements for the total number of levels implanted with LLIF and ALIF cages at baseline and at different postoperative timepoints (Table 3). Among the LLIF-only levels, the mean postoperative global lumbar lordosis was 44.86 ± 12.14 degrees and 46.09 ± 10.66 degrees at 12 and 24 months, respectively. Segmental lumbar lordosis was 32.76 ± 15.49 degrees and 27.47 ± 14.35 degrees at 12 and 24 months, respectively. Mean postoperative disc height was 9.45 ± 2.15 mm and 9.88 ± 2.32 mm at 12 and 24 months, respectively. Among the ALIF-only levels, the mean postoperative global lumbar lordosis was 46.96 ± 15.40 degrees and 38.80 ± 16.76 degrees at 12 and 24 months, respectively. Segmental lumbar lordosis was 31.73 ± 9.74 degrees and 24.58 ± 6.79 degrees at 12 and 24 months, respectively. Mean postoperative disc height was 11.02 ± 1.54 mm and 11.65 ± 1.05 mm at 12 and 24 months, respectively.

No intraoperative complications were reported. Of the post-operative adverse events, back pain was the most frequent event (LLIF—43.8%, ALIF—75.0%, Combined—50.0%). Other complications in the cohort included sensory dysfunction (LLIF—21.9%, ALIF—25.0%, Combined—50%), radiculopathy (LLIF—21.9%, ALIF—75%, Combined—25%), and stiffness (LLIF—18.8%, ALIF—25%, Combined—25%). Only one patient (2.1%) in the combined cohort required a revision to address ALIF cage migration. Reoperation (without revision) was reported in one LLIF-only patient (2.1%) due to decompression for right L5 nerve stenosis. No serious adverse events or deaths were reported (Table 4).

### 3.3. Within-Group Differences in PROMs

**Figure 2:**
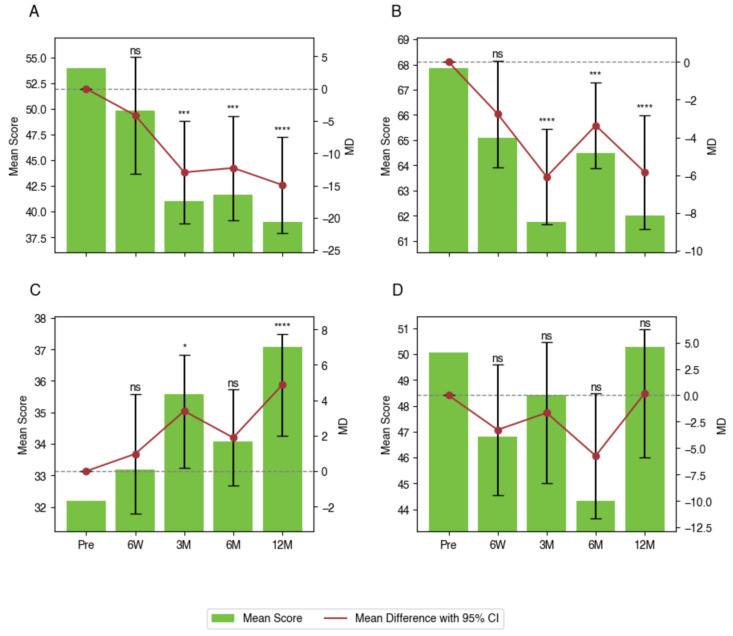
LMM analysis of peri-operative PROMs. (**A**) ODI, (**B**) PROMIS Pain Interference, (**C**) PROMIS Physical Function, and (**D**) PROMIS Depression. Significance levels: * if *p* < 0.05; *** if *p* < 0.001; **** if *p* < 0.0001.

Findings from the LMM analysis revealed statistically significant improvements across several PROMs compared to pre-operative baseline values. For ODI, significant improvements were observed at 3 months (MD −13.0 points with 95% CI −20.9 to −5.0 points, *p* < 0.001), 6 months (MD −12.3 points with 95% CI −20.4 to −4.2 points, *p* < 0.001), and 12 months (MD −14.9 points with 95% CI −22.4 to −7.5 points, *p* < 0.001). PROMIS Pain Interference scores demonstrated significant reductions compared to baseline values, with improvements at 3 months (MD −6.1 points with 95% CI −8.6 to −3.6 points, *p* < 0.001), 6 months (MD −3.4 points with 95% CI −5.6 to −1.1 points, *p* < 0.001), and 12 months (MD −5.8 points with 95% CI −8.9 to −2.8 points, *p* < 0.001). For PROMIS Physical Function scores, significant improvements were observed after 3 months (MD +3.4 points with 95% CI 0.2 to 6.6 points, *p* = 0.032) and 12 months (MD +4.9 points with 95% CI 2.0 to 7.8 points, *p* < 0.001). Improvements after 6 months (MD +1.9 points with 95% CI −0.8 to 4.6 points, *p* = 0.362) were observed but did not reach statistical significance. PROMIS Depression scores showed mixed results, with reductions observed after 6 months (MD −5.7 points with 95% CI −11.7 to −0.2 points, *p* = 0.065) that did not reach statistical significance. After 12 months, scores returned near-baseline levels (MD +0.2 points with 95% CI −5.9 to 6.3 points, *p* = 0.999). These findings indicate that the greatest benefits were observed within the first 12 months post-operatively, with significant improvements across multiple instruments (Table 5, Figure 2).

## 4. Discussion

This study evaluated the clinical and radiographic outcomes for 48 patients undergoing LLIF or ALIF using a novel titanium interbody cage system for the treatment of lumbar DSD. To our knowledge, this is the first study to present the outcomes of fusion using a porous titanium cage with combined microrough and nanostructure technology in LLIF, ALIF, and combined cohorts. Our findings demonstrated excellent radiographic fusion rates and significant improvements in patient-reported outcomes, particularly in terms of pain and physical function. Fusion rates were highest in the LLIF-only cohort, with 90.3% of treated levels achieving fusion by the final follow-up at ≥12 months. PROMs analysis of the whole cohort showed substantial improvements in ODI, PROMIS Pain Interference, and PROMIS Physical Function scores at multiple postoperative timepoints, compared with their preoperative baseline. Notably, adverse events and complications were minimal, with no intraoperative complications and a low rate of revision surgeries.

Although the combined LLIF and ALIF cohort demonstrated a lower fusion rate than the other cohorts, this may be attributed to increased case complexity, as demonstrated by the greater number of levels operated, longer operative times and lengths of stays, and greater estimated blood loss. The high fusion rates observed in this study among the other cohorts align with previous research on porous titanium interbody devices, which have demonstrated enhanced osteointegration and long-term stability [26,27,28]. The cage’s porous microroughened surface, combined with nanoscale texturing, likely contributed to the successful fusion outcomes observed herein. Compared to traditional PEEK cages, this titanium-based cage may offer clinically equivalent or superior osteoconductive properties, promoting bone growth and reducing the risk of non-union [28,29,30,31]. The differences in fusion rates between the LLIF and ALIF cohorts may reflect biomechanical differences in load distribution and challenges that are unique to the L5/S1 level [32,33]. While most patients achieved fusion at or after the 6-month follow-up point, two patients were classified as fused before 3 months had passed. These early assessments were made based on bridging bone without lucency and were confirmed with later imaging, since early assessments of fusion can be susceptible to misclassification [34]. Although radiographic parameters such as anterior/posterior disc height and segmental lordosis showed relative stability over time, some variability was observed, particularly at early postoperative timepoints. This may reflect mild cage settling, patient positioning variability, or the limitations inherent in plain radiograph measurement methods. Given these factors, we do not interpret these parameters in isolation as definitive indicators of fusion, but rather as a supportive context that complements Bridwell fusion grading. Future prospective studies incorporating CT or motion analysis may more precisely quantify fusion-related biomechanical stability.

In addition to fusion rates, PROMs provided valuable insights into the patient’s functional status following surgery. Significant reductions in ODI and PROMIS Pain Interference scores at 3, 6, and 12 months suggest that these cage systems effectively addressed pain and the functional limitations associated with lumbar degenerative disease. The improvements in PROMIS Physical Function scores further support the notion that these devices facilitate early recovery and sustained functional gains. PROMIS Depression scores did not demonstrate sustained improvements, which may indicate either that psychosocial factors remain a critical consideration in postoperative recovery or that baseline levels indicated little psychological distress. While statistical significance was achieved across the various outcome measures, tangible improvements in patient quality of life remain one of the key success metrics. Our data indicate that these improvements are both statistically robust and clinically meaningful, with reductions in pain and disability translating to better functional outcomes and patient satisfaction scores maintained at 12 months postoperatively. This study also highlighted the safety profile of the novel cage system. No intraoperative complications were reported, and the rates of revision and reoperation were low across all cohorts. Radiographic complications such as subsidence and cage migration were observed, but these occurred at levels consistent with previously published findings in the literature [26,27].

Despite the evolving landscape of minimally invasive spine surgery, there is still little consensus on the best treatment for DSD. Minimally invasive spinal surgery has gained increasing traction over the past two decades as a means to reduce surgical morbidity, improve recovery times, and enhance patient outcomes [35,36,37]. Techniques such as LLIF and ALIF are representative of this shift, offering indirect decompression and stabilization while avoiding the disruption of posterior musculature and neural elements [38,39]. These approaches are particularly valuable in treating DSD, where maintaining spinal alignment and achieving fusion are critical to long-term success.

Advances in cage technology, including the use of porous and nanostructured materials, further support this paradigm shift by promoting osteointegration and reducing complications such as subsidence and migration [15,17]. This cage system’s unique combination of microroughened and nanostructured titanium surfaces aims to facilitate both initial stability and long-term fusion through enhanced bone–implant interaction. The cage’s design, with its optimized pore size and an elastic modulus tailored to mimic native bone properties, offers a promising solution to some of the limitations seen with traditional interbody devices [13,14,16,18]. In addition to the implant’s surface characteristics, the type of bone graft material used may also influence fusion outcomes. In our series, a variety of graft materials, including autograft, allograft, bone marrow aspirate, and synthetic options, were employed as detailed in Table 2. While this reflects real-world practice, this study was not designed to assess the independent contribution made by each type of graft. However, a previous study by Zeitouni et al. found no difference in lumbar fusion outcomes between bone graft types [40].

As the adoption of advanced interbody technologies increases, it is imperative to evaluate their performance across diverse patient populations and clinical contexts. Several studies have demonstrated similar or superior clinical and radiographic outcomes in 3D-printed titanium cages compared to PEEK [41,42,43]. Other studies have demonstrated that healthcare utilization and hospital costs are similar between PEEK and 3D-printed titanium cages [44]. Our results suggest that these titanium interbody devices may offer a viable alternative to traditional PEEK or static titanium cages, and additional comparative analyses of cage materials are warranted in the future.

### Limitations

Several limitations of this study must be acknowledged. The retrospective, single-center design limits the generalizability of the findings. Because the study was retrospective and included all eligible patients during the study period, no a priori power calculations were performed. As such, the possibility of a type-II error cannot be excluded, and larger, adequately powered prospective studies are needed to validate these findings. Additionally, this study lacked a contemporaneous control group of patients undergoing alternative surgical techniques or conservative management, which limits direct comparisons and the generalizability of the findings. Future prospective studies with randomized or matched control cohorts are warranted to validate these results and assess comparative effectiveness. In addition, the evaluation of fusion relied primarily on standing lateral radiographs, using the Bridwell interbody fusion grading system. CT scans were incorporated when available, but these were not obtained systematically, due to the retrospective nature of the study. As such, fusion assessment may be limited by the inherent constraints of X-ray imaging, including its lower sensitivity for early or subtle osseous bridging compared to CT. Additionally, a single observer was used for making the assessments. While this minimized interobserver variability, the use of plain radiographs alone is subject to interpretive bias, and future studies employing multiple independent evaluators and advanced imaging modalities may improve the reliability of fusion assessment.

There was also missingness in the follow-up PROMs data after 24 months, precluding extensive analysis beyond 12 months. Missingness at other timepoints was addressed with imputation techniques when applicable, and this could introduce some uncertainty into the analysis; therefore, the possibility of residual bias cannot be excluded. Additionally, although a variety of validated PROMs exist, our institution primarily records ODI and PROMIS scores in the electronic health record. As a result, our analysis was limited to these measures. Future studies incorporating larger, multicenter cohorts with a comparator group and additional PROMs will aid in validating these findings and further defining the role of novel cage technologies in lumbar spine surgery to treat DSD.

## 5. Conclusions

This study demonstrated that the present novel titanium interbody cage system is a safe and effective option for achieving high fusion rates and offers significant improvements in pain and function in patients undergoing LLIF and ALIF for lumbar DSD. The current study showed improvements in clinical and radiographic outcomes, satisfactory radiographic fusion, and minimal adverse events. While our results are promising, further research is needed to confirm the long-term clinical benefits and explore the broader applicability of this technology in minimally invasive spine surgery by comparing its efficacy with other commonly used fusion devices.

## Figures and Tables

**Table 1:** Demographic and baseline characteristics.

Characteristic ^a^	LLIF (n = 32)	LLIF + ALIF (n = 12)	ALIF (n = 4)	Total (n = 48)
Overall N patients (%)	32 (66.7)	12 (25)	4 (8.3)	48 (100)
Gender, n (%)				
Female	20 (62.5)	7 (58.3)	3 (75.0)	30 (62.5)
Male	12 (37.5)	5 (41.7)	1 (25.0)	18 (37.5)
Age (years)				
Mean ± SD	60.3 ± 10.5	62.3 ± 12.4	42.8 ± 10.9	59.4 ± 12.0
Median	61	65	47	60.5
Range (Min, Max)	35, 79	36, 80	27, 50	27, 80
BMI (kg/m^2^)				
Mean ± SD	29.6 ± 4.5	29.9 ± 4.8	32.1 ± 9.3	29.9 ± 5.0
Median	29.0	28.4	32.4	29.0
Range (Min, Max)	20.9, 39.3	21.6, 37.0	20.4, 43.2	20.4, 43.2
Preop. Smoking History, n (%)				
Current smoker	3 (9.4)	1 (8.3)	2 (50.0)	6 (12.5)
Previous smoker	6 (18.8)	6 (50.0)	-	12 (25.0)
Never smoker	23 (71.9)	5 (41.7)	2 (50.0)	30 (62.5)
Comorbidities at Time of Surgery, n (%)				
COPD	1 (3.1)	-	-	1 (2.1)
Diabetes	8 (25.0)	2 (16.7)	-	10 (20.8)
Obesity	4 (12.5)	3 (25.0)	3 (75.0)	10 (20.8)
Osteoporosis ^b^	1 (3.1)	-	-	1 (2.1)
Steroid Usage	-	1 (8.3)	-	1 (2.1)
Previous Spine Surgery				
Lumbar Fusion	7 (21.9)	1 (8.3)	1 (25.0)	9 (18.8)
Lumbar Other	5 (15.6)	3 (25.0)	-	8 (16.7)
Cervical Spine	-	3 (25.0)	-	3 (6.3)
Length of Follow-up Time (months)				
Mean ± SD	11.0 ± 5.7	15.4 ± 4.9	10.0 ± 6.4	12.0 ± 5.8
Median	11.0	13.0	11.5	12.0
Range (Min, Max)	2.0, 25.0	11.0, 24.0	1.0, 16.0	1.0, 25.0
Patient Diagnosis, n (%) ^c^				
Disc degeneration	11 (34.4)	4 (33.3)	2 (50.0)	17 (35.4)
Radiculopathy	3 (9.4)	1 (8.3)	-	4 (8.3)
Stenosis	12 (37.5)	7 (58.3)	1 (25.0)	20 (41.7)
Neurogenic claudication	4 (12.5)	5 (41.7)	1 (25.0)	10 (20.8)
Lumbar spondylosis	9 (28.1)	7 (58.3)	-	16 (33.3)
Spondylolisthesis	21 (65.6)	4 (33.3)	2 (50.0)	27 (56.3)
Pseudarthrosis	2 (6.3)	-	-	2 (4.2)
Adjacent segment disease	3 (9.4)	1 (8.3)	-	4 (8.3)
Off-label: Osteomyelitis ^d^	1 (3.1)	-	-	1 (2.1)

^a^ Analysis and % calculated based on N = no. of patients in the subgroups and total no. of patients. ^b^ Osteoporosis was noted since comorbidity, severity, or means of diagnosis was not reported, hence this was not considered off-label. ^c^ Patients had a combination of multiple diagnoses and are not mutually exclusive. ^d^ Osteomyelitis was a secondary diagnosis in a progressive stenosis patient. Reported as off-label for conservative purposes.

**Table 2:** Procedural characteristics.

Characteristic	LLIF (n = 32)	LLIF + ALIF (n = 12)	ALIF (n = 4)	Total (n = 48)
Retroperitoneal Surgical Approach, n (%)				
Anterior to Psoas (ATP)	6 (18.8)	6 (50.0)	-	12 (25.0)
Transpsoas	26 (81.3)	6 (50.0)	4 (100.0)	36 (75.0)
Operation Duration, n (%)				
≤2 h	5 (15.6)	-	-	5 (10.4)
2–3 h	8 (25.0)	1 (8.3)	-	9 (18.8)
3–4 h	6 (18.8)	-	-	6 (12.5)
>4 h	13 (40.6)	11 (91.7)	3 (75.0)	27 (56.3)
Estimated Blood Loss (cc)				
Mean	210.9	420.8	175.0	260.4
SD	225.8	229.1	86.6	235.0
Median	150.0	425.0	200.0	200.0
Min, Max	0.0, 850.0	50.0, 900.0	50.0, 250.0	0.0, 900.0
Length of Hospital Stay, n (%)				
1 day	3 (9.4)	-	-	3 (6.3)
≤5 days	23 (71.9)	9 (75.0)	3 (75.0)	35 (72.9)
6–10 days	6 (18.8)	2 (16.7)	1 (25.0)	9 (18.8)
>10 days	-	1 (8.3)	-	1 (2.1)
Number of Levels Cages Implanted, n (%)				
1	24 (75.0)	-	4 (100.0)	28 (58.3)
2	5 (15.6)	6 (50.0)	-	11 (22.9)
3	3 (9.4)	5 (41.7)	-	8 (16.7)
4	-	1 (8.3)	-	1 (2.1)
Vertebral Levels Implanted ^a^, n (%)				
L1/L2	-	1 (8.3)	-	1 (2.1)
L2/L3	7 (21.9)	2 (16.7)	-	9 (18.8)
L3/L4	15 (46.9)	6 (50.0)	-	21 (43.8)
L4/L5	20 (62.5)	10 (83.3)	-	30 (62.5)
L5/S1	1 (3.1)	12 (100.0)	4 (100.0)	17 (35.4)
Supplemental Fixation Used, n (%)				
Pedicle screws	28 (87.5)	9 (75.0)	4 (100.0)	41 (85.4)
None	3 (9.4)	1 (8.3)	-	4 (8.3)
Missing	1 (3.1)	2 (16.7)	-	3 (6.3)
Graft Material ^b^, n (%)				
Autograft (local bone)	4 (12.5)	3 (25.0)	-	7 (14.6)
Autograft (iliac crest)	13 (40.6)	8 (66.7)	2 (50.0)	23 (47.9)
Allograft	30 (93.8)	12 (100.0)	4 (100.0)	46 (95.8)
BMA	30 (93.8)	12 (100.0)	2 (50.0)	44 (91.7)
chronOS	1 (3.1)	-	-	1 (2.1)
ViviGen	1 (3.1)	-	1 (25.0)	2 (4.2)
Hydroxyapatite synthetic graft	1 (3.1)	-	-	1 (2.1)

^a^ Patients had implants at more than one level, and the levels were not mutually exclusive. ^b^ Patients received multiple graft materials, and the graft materials were not mutually exclusive.

**Table 3:** Radiographic characteristics change ^2^ from baseline according to cage types.

Characteristic	Preop. Baseline	6 Weeks Change	3 Months Change	6 Months Change	12 Months Change	24 Months Change
LLIF ^1^ (n, n missing)	60, 2	46, 16	49, 13	42, 20	42, 20	23, 39
Global Lumbar Lordosis (°)	43.52 ± 13.19	−0.49 ± 7.82	0.55 ± 13.30	1.73 ± 7.56	1.31 ± 10.49	0.20 ± 8.08
Segmental Lumbar Lordosis (°)	31.42 ± 16.52	−0.82 ± 12.23	0.37 ± 12.25	2.21 ± 7.45	1.50 ± 12.57	−4.08 ± 14.96
Anterior Disc Space Height (mm)	8.15 ± 3.66	3.96 ± 3.60	4.75 ± 4.29	4.08 ± 4.33	4.16 ± 3.70	3.85 ± 4.23
Posterior Disc Space Height (mm)	4.78 ± 2.15	2.79 ± 2.78	3.33 ± 2.49	3.01 ± 2.09	2.74 ± 2.75	2.95 ± 2.56
Mean Disc Space Height (mm)	6.16 ± 2.61	3.37 ± 2.70	4.04 ± 3.08	3.54 ± 2.90	3.45 ± 2.95	3.40 ± 3.15
Foraminal Height (mm)	16.53 ± 4.83	2.64 ± 4.73	2.71 ± 4.45	2.47 ± 5.69	2.44 ± 3.82	2.86 ± 4.36
ALIF ^1^ (n, n missing)	16, 0	12, 4	13, 3	11, 5	9, 7	4, 12
Global Lumbar Lordosis (°)	42.19 ± 15.10	0.66 ± 9.14	1.53 ± 13.77	3.21 ± 5.41	8.06 ± 12.06	0.78 ± 2.72
Segmental Lumbar Lordosis (°)	24.38 ± 12.48	4.93 ± 8.58	6.24 ± 6.30	4.34 ± 5.67	8.20 ± 10.80	4.40 ± 4.77
Anterior Disc Space Height (mm)	8.23 ± 3.97	8.38 ± 3.79	8.50 ± 3.29	8.34 ± 3.78	8.72 ± 2.11	9.20 ± 4.10
Posterior Disc Space Height (mm)	4.10 ± 1.61	3.84 ± 1.99	3.95 ± 1.50	3.80 ± 1.93	3.81 ± 1.56	4.83 ± 1.56
Mean Disc Space Height (mm)	6.16 ± 2.61	6.11 ± 2.37	6.22 ± 1.90	6.07 ± 2.33	6.27 ± 1.47	7.01 ± 2.17
Foraminal Height (mm)	12.14 ± 4.43	3.89 ± 3.67	2.76 ± 4.22	4.00 ± 3.37	4.42 ± 3.41	7.10 ± 2.63

^1^ n is the number of levels with available data. ^2^ Change calculated from baseline score; the − sign only indicates directionality.

**Table 4:** Adverse events and radiographic outcomes.

Characteristic	LLIF (n = 32)	LLIF+ALIF (n = 12)	ALIF (n = 4)	Total (n = 48)
AE Severity ^a^, n (%)				
Mild	34 (106.3)	17 (141.7)	5 (125.0)	56 (116.7)
Moderate	10 (31.3)	5 (41.7)	2 (50.0)	17 (35.4)
Serious (SAE)	-	-	-	-
n missing	1 (3.1)	-	-	1 (2.1)
AE Relatedness to Device (Cage), n (%)				
Not Related	40 (125.0)	16 (133.3)	4 (100.0)	60 (125.0)
Probably Related	-	2 (16.7)	-	2 (4.2)
Possibly Related	1 (3.1)	2 (16.7)	3 (75.0)	6 (12.5)
Definitely Related	3 (9.4)	2 (16.7)	-	5 (10.4)
n missing	1 (3.1)	-	-	1 (2.1)
AE Relatedness to Procedure, n (%)				
Not Related	14 (43.8)	3 (25.0)	-	17 (35.4)
Probably Related	3 (9.4)	3 (25.0)	1 (25.0)	7 (14.6)
Possibly Related	23 (71.9)	14 (116.7)	6 (150.0)	43 (89.6)
Definitely Related	3 (9.4)	2 (16.7)	-	5 (10.4)
n missing	1 (3.1)	-	-	1 (2.1)
Revision/Reoperation Summary, n (%)				
Reoperation– Right L5 Nerve Decompression by Transforaminal Approach for Restenosis	1 (3.1)	-	-	1 (2.1)
Revision– L5/S1 ALIF Cage Replaced by Same 3D-printed titanium ALIF Cage for Cage Migration	-	1 (8.3) ^b^	-	1 (2.1)
Radiographic Complications, n (%)				
Non-union (≥ 12 months ^c^)	3 (9.4)	4 (33.3)	-	7 (14.6)
Cage Subsidence (grades 1, 2, and 3) (at any timepoint)	6 (18.8)	2 (16.7)	-	8 (16.7)
Cage Migration (at any timepoint)	1 (3.1)	2 (16.7)	1 (25.0)	4 (8.3)
Spondylolisthesis (at any timepoint)	1 (3.1)	-	-	1 (2.1)

^a^ Patients had multiple complications and were not mutually exclusive. All clinical complications at different timepoints are included. ^b^ Involved ALIF cage in the combined cohort. ^c^ Patients with no radiographic fusion at ≥12 months are considered non-union.

**Table 5:** Summary of the LMM results.

PROM	Timepoint	Mean Score (95% CI)	Mean Difference (95% CI)	*p*-Value
ODI	Pre-Op	54.0 (47.1 to 60.9)	-	-
	6 Weeks	49.8 (43.6 to 56.0)	−4.1 (−13.2 to 4.9)	1.0000
	3 Months	41.0 (35.8 to 46.2)	−13.0 (−20.9 to −5.0)	0.0002
	6 Months	41.7 (36.4 to 47.0)	−12.3 (−20.4 to −4.2)	0.0005
	12 Months	39.0 (34.3 to 43.7)	−14.9 (−22.4 to −7.5)	<0.0001
PROMIS Pain Interference	Pre-Op	67.9 (65.7 to 70.0)	-	-
	6 Weeks	65.1 (63.0 to 67.2)	−2.8 (−5.6 to 0.1)	0.0606
	3 Months	61.8 (59.9 to 63.7)	−6.1 (−8.6 to −3.6)	<0.0001
	6 Months	64.5 (62.8 to 66.2)	−3.4 (−5.6 to −1.1)	0.0008
	12 Months	62.0 (59.7 to 64.3)	−5.9 (−8.9 to −2.8)	<0.0001
PROMIS Physical Function	Pre-Op	32.2 (29.8 to 34.6)	-	-
	6 Weeks	33.2 (30.7 to 35.6)	1.0 (−2.4 to 4.4)	1.0000
	3 Months	35.6 (33.3 to 37.9)	3.4 (0.2 to 6.6)	0.0317
	6 Months	34.1 (32.2 to 35.9)	1.9 (−0.8 to 4.6)	0.3617
	12 Months	37.1 (35.1 to 39.1)	4.9 (2.0 to 7.8)	0.0001
PROMIS Depression	Pre-Op	50.1 (46.2 to 53.9)	-	-
	6 Weeks	46.8 (43.8 to 49.8)	−3.3 (−9.5 to 3.0)	0.8641
	3 Months	48.4 (44.8 to 52.0)	−1.7 (−8.3 to 5.0)	1.0000
	6 Months	44.3 (41.7 to 47.0)	−5.7 (−11.7 to 0.2)	0.0653
	12 Months	50.3 (47.4 to 53.1)	0.2 (−5.9 to 6.3)	1.0000

## Data Availability

The data presented in this study are available upon request from the corresponding author.

## Abbreviations

The following abbreviations are used in this manuscript:

LLIF	Lateral lumbar interbody fusion
ALIF	Anterior lumbar interbody fusion
ODI	Oswestry Disability Index
PROMIS	Patient-Reported Outcomes Measurement Information System
DSD	Degenerative spine disease
PEEK	Polyether ether ketone
PROMs	Patient-reported outcome measures
STROBE	Strengthening the reporting of observational studies in epidemiology
TRIPOD+AI	Transparent reporting of a multivariable prediction model for individual prognosis or diagnosis + artificial intelligence
AE	Adverse events
LMM	Linear mixed-effects model
